# Resonance assignment and secondary structure of DbpA protein from the European species, *Borrelia afzelii*

**DOI:** 10.1007/s12104-021-10039-2

**Published:** 2021-08-06

**Authors:** Libor Hejduk, Petr Rathner, Martin Strnad, Libor Grubhoffer, Jan Sterba, Ryan O. M. Rego, Norbert Müller, Adriana Rathner

**Affiliations:** 1 Faculty of Science, University of South Bohemia, Branišovská 1760, 37005 České Budějovice, Czech Republic; 2 Institute of Parasitology, Biology Centre, Czech Academy of Sciences, Branišovská 31, 37005 České Budějovice, Czech Republic; 3 Institute of Inorganic Chemistry, Johannes Kepler University, Altenbergerstraße 69, 4040 Linz, Austria; 4 Institute of Organic Chemistry, Johannes Kepler University, Altenbergerstraße 69, 4040 Linz, Austria; 5 Institute of Analytical Chemistry, University of Vienna, Währingerstraße 38, 1090 Vienna, Austria

**Keywords:** NMR resonance assignment, Decorin-binding proteins, *Borrelia afzelii*

## Abstract

Decorin binding proteins (Dbps) mediate attachment of spirochetes in host organisms during the early stages of Lyme disease infection. Previously, different binding mechanisms of Dbps to glycosaminoglycans have been elucidated for the pathogenic species *Borrelia burgdorferi* sensu stricto and *B. afzelii.* We are investigating various European *Borrelia* spirochetes and their interactions at the atomic level using NMR. We report preparative scale recombinant expression of uniformly stable isotope enriched *B. afzelii* DbpA in *Escherichia coli*, its chromatographic purification, and solution NMR assignments of its backbone and sidechain ^1^H, ^13^C, and ^15^N atoms. This data was used to predict secondary structure propensity, which we compared to the North American *B. burgdorferi* sensu stricto and European *B. garinii* DbpA for which solution NMR structures had been determined previously. Backbone dynamics of DbpA from *B. afzelii* were elucidated from spin relaxation and heteronuclear NOE experiments. NMR-based secondary structure analysis together with the backbone dynamics characterization provided a first look into structural differences of *B. afzelii* DbpA compared to the North American species and will serve as the basis for further investigation of how these changes affect interactions with host components.

## Biological context

*Borrelia burgdorferi* sensu lato (s.l.) complex of genospecies is the causative agent of Lyme disease, the most common tick-borne disease in Europe and North America. The Lyme disease manifestation includes tissue tropism related to colonization by particular *Borrelia* genospecies. For instance, *B. burgdorferi* sensu stricto (s.s.) preferentially colonises joints while *B. garinii*, whose infection leads to neuroborreliosis, prefers neural tissues (Wang et al., 1999). The development of Lyme disease, primarily during the early phase, proceeds by invasion and adhesion of bacteria to different structures in the host organism. The outer surface of *Borrelia* is coated with various proteins including adhesins, which mediate attachment to cell surface proteins or other molecules in the extracellular matrix.

Decorin binding proteins (Dbps) are important adhesins exposed on the surface of bacteria from the *B. burgdorferi* s.l. complex. Dbps bind collagen-associated protein decorin through glycosaminoglycan (GAG) chain attached to the decorin (Fischer et al., 2003). Decorin is a glycoprotein highly abundant in the connective tissues associated with collagen fibres. Decorin is modified with various GAG chains depending on its presence in different tissues. DbpA and DbpB, two homologous Dbps, have been described as important factors for *Borrelia* virulence and host colonization. According to previous research (Shi et al., 2008), cooperation of both homologs in binding to decorin is necessary for tissue colonization. DbpA is species variable in its amino acid sequence, whereas DbpB is more conserved. The sequence similarity of DbpA across the genospecies is above 58% in contrast to DbpB which lies above 96% (Roberts et al., 1998; Fig. 3). Based on the sequence identity, DbpA variants also differ in their binding affinity to different GAGs attached to decorin (Lin et al., 2014). Combining these aspects – tissue tropism of bacteria and structural variability of adhesins including Dbps, DbpA-GAG interaction variations are acknowledged to have a considerable effect on the pathogenicity of *Borrelia* genospecies. Characterization of DbpA from various *B. burgdorferi* s.l. by solution NMR spectroscopy, i. e. under near-native conditions will help to establish a starting point in deciphering exact interaction schemes between DbpA of *B. afzelii* and of other species and small GAGs which have been studied only in North American *Borrelia* strains so far. For comprehensive understanding of these relatively weak interactions assessment of the protein backbone dynamics is crucial.

## Methods and experiments

### Cloning, expression, and purification of DbpA

The gene coding sequence for DbpA from *B. afzelii* (strain A91) without the transmembrane part of the protein was cloned into pQE30 plasmid, which includes the sequence for His_6_ tag directly attached to N-terminus of the protein (full amino acid sequence of the recombinant protein can be found in Fig. 2a). The construct was transformed into *E. coli* M15 (pREP4) strain. 20 mL Lysogeny Broth (LB) media was inoculated by the cells and grown for 12 h at 37 °C as an overnight culture. The culture was used in a dilution 1:100 for inoculation of fresh LB medium in a volume of 250 mL. The cell culture was cultivated at 37 °C with shaking at 200 rpm and after the optical density (OD 600 nm) reached 0.7, the cells were centrifuged at 3000 × g for 30 min. The pelleted cells were resuspended in the same volume of M9 minimal media supplemented with ^15^N (> 98%, Cambridge Isotope Laboratories, Inc.) ammonium sulphate (1.5 g/l) and uniformly ^13^C (> 99%, Cambridge Isotope Laboratories, Inc.) labelled glucose (2 g/l). The temperature was lowered to 25 °C, after 1 h the cells were induced by 1 mM IPTG and incubated for 18 h at 25 °C with shaking at 200 rpm. The cells were harvested and resuspended in 10 mL of buffer A (buffer A: 20 mM Tris, 200 mM NaCl, pH 7.2; buffer B: 20 mM Tris, 200 mM NaCl, 500 mM imidazole, pH 7.2) with Halt Protease inhibitor mix (Thermo Fisher Scientific). Cells were disrupted using French press (Stansted Fluid Power Ltd.) at approx. 120 MPa and lysate was centrifuged in an ultracentrifuge at 70,000 × g for 1 h. The first purification step was Ni^2+^ affinity chromatography performed on 5 mL HisTrap HP column (Cytiva). The lysate was directly applied to the column equilibrated with Buffer A. Non-specifically bound proteins were washed out by step of 12% buffer B. DbpA was received within the gradient elution of 12% – 100% of buffer B. The fractions containing DbpA were concentrated by Amicon Ultra 10 K filter columns. In the second step, the concentrated sample was purified with size exclusion chromatography on SuperDex 75 10/300 GL (Cytiva) using a constant flow of 0.2 mL/min of running buffer (50 mM KH_2_PO_4_, 200 mM NaCl, pH 7.2).

### Nuclear magnetic resonance spectroscopy

All NMR experiments were recorded on a 700 MHz Avance III spectrometer with an Ascend magnet and TCI cryoprobe (manufactured in 2011 by Bruker). Uniformly ^15^N, ^13^C labelled DbpA was measured in 20 mM KH_2_PO_4_, pH 6.0, 10% D_2_O at 470 µM concentration enriched with 1/7 of the sample volume of stock solution of Protease cOmplete® Mini inhibitors cocktail, EDTA free (stock solution contained 1 tablet/1.5 mL; Roche).

To determine the ideal temperature for further measurements, a set of ^15^N TROSY-HSQC experiments in thermal gradient was performed at temperatures ranging from 288 to 315 K and back (3 K steps). Best signal-to-noise ratio and peak dispersion were observed at 313 K.

Spectra recorded for backbone assignment comprised: ^1^H-^15^N HSQC, ^1^H-^13^C HSQC, ^15^N TOCSY-HSQC, ^1^H-^15^N TROSY-HSQC, HNCO, HNCA, HNHA, HNCACB, CBCA(CO)NH. In addition to mentioned experiments, H(CCO)NH, CC(CO)NH, (H)CCH-TOCSY and HCCH-COSY were recorded to assign the sidechain atoms. (Bax et al., 1990; Sattler, 1999; Grzesiek et al., 1993; Vuister and Bax, 1993) All spectra were processed using Topspin 3.6.1 (Bruker). The resonance assignment of backbone and side chain resonances of DbpA was accomplished manually in CARA program (Keller, 2004). Secondary structure propensity was analysed by online prediction service TALOS-N based on calculations of backbone torsion angles ϕ, ψ and sidechain torsion angle χ from experimentally measured chemical shifts (Shen and Bax, 2013).

Local dynamics was assessed with T_1_, T_2_ and heteronuclear ^15^N {^1^H} NOEs values. For ^15^N T_1_ relaxation times, 2D phase sensitive ^1^H-^15^N HSQC using inversion recovery with PEP (Preservation of Equivalent Pathways) sensitivity improvement was recorded using the pulse program hsqct1etf3gpsi (inversion recovery delays were following: 10, 50, 100, 200, 300, 400, 500, 600, 700, 800, 900, 1000, 1100 and 1200 ms) (Canavagh et al., 2007). ^15^N T_2_ relaxation times were determined in an analogous way to T_1_ times using a version of the previously mentioned 2D experiment specific to T_2_ relaxation times, hsqct2etf3gpsi (delays of 5, 10, 15, 20, 25, 30, 35, 40, 50, 60, 70, 80, 90, 100 and 120 ms). Backbone heteronuclear NOEs were obtained from the phase sensitive gradient-enhanced 2D ^1^H-^15^N HSQC using PEP sensitivity improvement (hsqcnoef3gpsi Bruker pulse program) with a saturation time (d1 parameter) of 1 s. All spectra were processed equally (with the same intensity scaling factor) and analyzed in NMRFAM-Sparky (Lee et al., 2015).

### Extent of assignments and data deposition

**Fig. 1:**
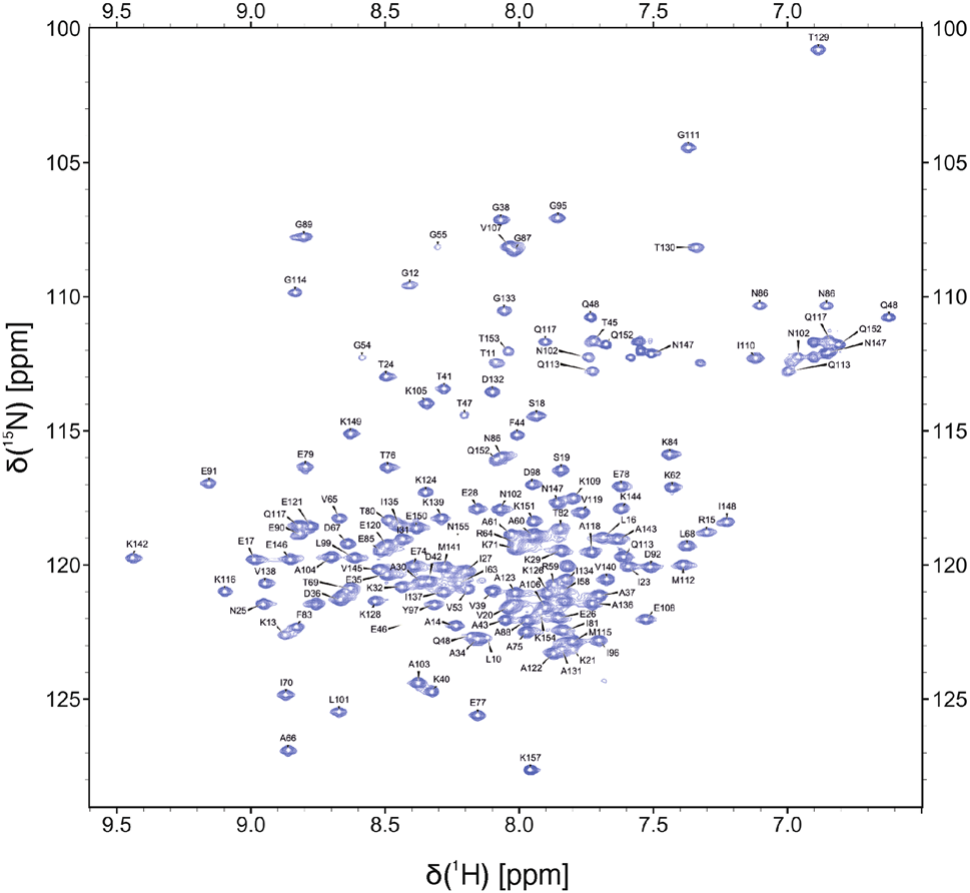
^1^H-^15^N HSQC spectrum of DbpA from *B. afzelii*. Peaks are labelled by a one letter code denoted with position of residue in the recombinant protein sequence. Figure was created using NMRFAM-Sparky (Lee et al., 2015)

The whole recombinant construct (including the N-terminal His_6_ tag and linker sequence) contains 157 residues from which 135 amino acids were at least partially assigned sequence specifically (Fig. 1). Assignments were deposited in BMRB under ID 50751. Unassigned remain the His_6_ tag, GS-linker and 14 residues from across the protein which makes the total extent of 90.6% assignment of the DbpA sequence (86% of all amino acids in the construct). We have assigned 91.1% of the backbone, 75.4% of side chains and 90.7% of ^1^H, ^15^N, ^13^Ca, ^13^Cb, ^13^CO, respectively (not taking into account the tag and linker residues). From the total of 22 unassigned residues in the protein (14 within the original DbpA sequence), 8 were located in ^1^H-^15^N HSQC spectra but could not be assigned unequivocally due to severe overlap in the center of the ^1^H-^15^N HSQC spectrum as well as lack of intensity for these systems in 3D spectra (e. g. ^15^N TOCSY-HSQC). Systems which were assigned with amino acid type and position in sequence also have most of the side chain atoms assigned.

**Fig. 2:**
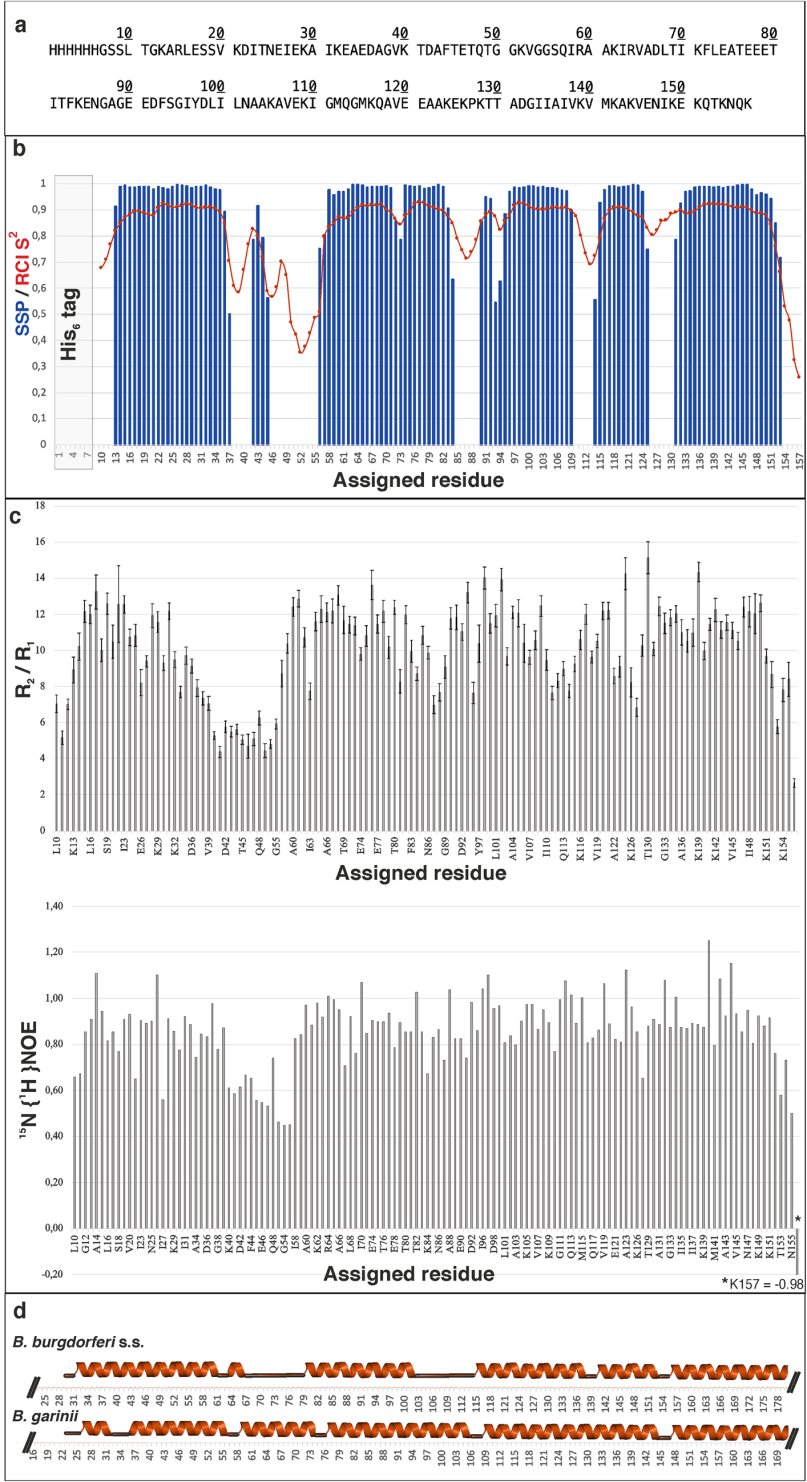
Secondary structure propensity and backbone dynamics of DbpA. **a**: Complete amino acid sequence of DbpA from *B. afzelii* including the N-terminal His_6_tag **b**: TALOS-N secondary structure propensity (SSP) of DbpA from backbone chemical shifts of all assigned residues. Blue bars represent the propensities of given amino acids to form an alpha helix. Residues with no value shown in the SSP plot were predicted to be random coil. The red line indicates the random coil index order parameter S^2^ **c**: R_2_/R_1_ spin relaxation rate ratios for backbone amides of all assigned residues (upper graph) and heteronuclear steady state ^15^N {^1^H} NOE values for all assigned amino acids (lower graph) **d**: Secondary structures of two DbpA protein homologs from North American *Borrelia* are plotted for comparison (DbpA from *B. burgdorferi* s.s.-PDB ID: 2MTC; DbpA from *B. garinii* – PDB ID: 2MTD; both in Morgan and Wang, 2015). Alignment of all data of DbpA with the secondary structures of other DbpAs in this graph is based on their sequential alignment using Clustal Omega (https://www.ebi.ac.uk/Tools/msa/clustalo/) which can be found in Fig. 3

Results from the TALOS-N secondary structure propensity prediction tool reveal that the secondary structure profile of European *B. afzelii* DbpA is generally similar to the DbpA solution NMR structures of two *Borrelia* species – *B. burgdorferi* s.s. (North America) and *B. garinii* (Europe) DbpAs (Fig. 2b, d). The longest loop (res. G38 – G55) of *B. afzelii* DbpA contains a small approx. one-turn alpha helix just like DbpA from *B. burgdorferi* s.s., whereas in the more sequentially related *B. garinii* DbpA one found a long alpha helix in the same place. The second substantial difference we find in the short loop (E85 – G89) region: in *B. garinii* DbpA there is an extended helix while in *B. burgdorferi* DbpA the disordered regions extend from T104 to S112. The secondary structure similarity of *B. burgdorferi* s.s. and *B. afzelii* DbpAs appears to be bigger than the one to *B. garinii* DbpA, although somewhat surprisingly, the sequence similarity behaves in the opposite way. These structural differences within DbpAs of different *Borrelia* species most likely mirror the difference in species specificity for various host tissues. It is also to be expected that these structural characteristics will be responsible for different affinities of DbpAs to various GAG chains across *Borrelia* species.

**Fig. 3:**
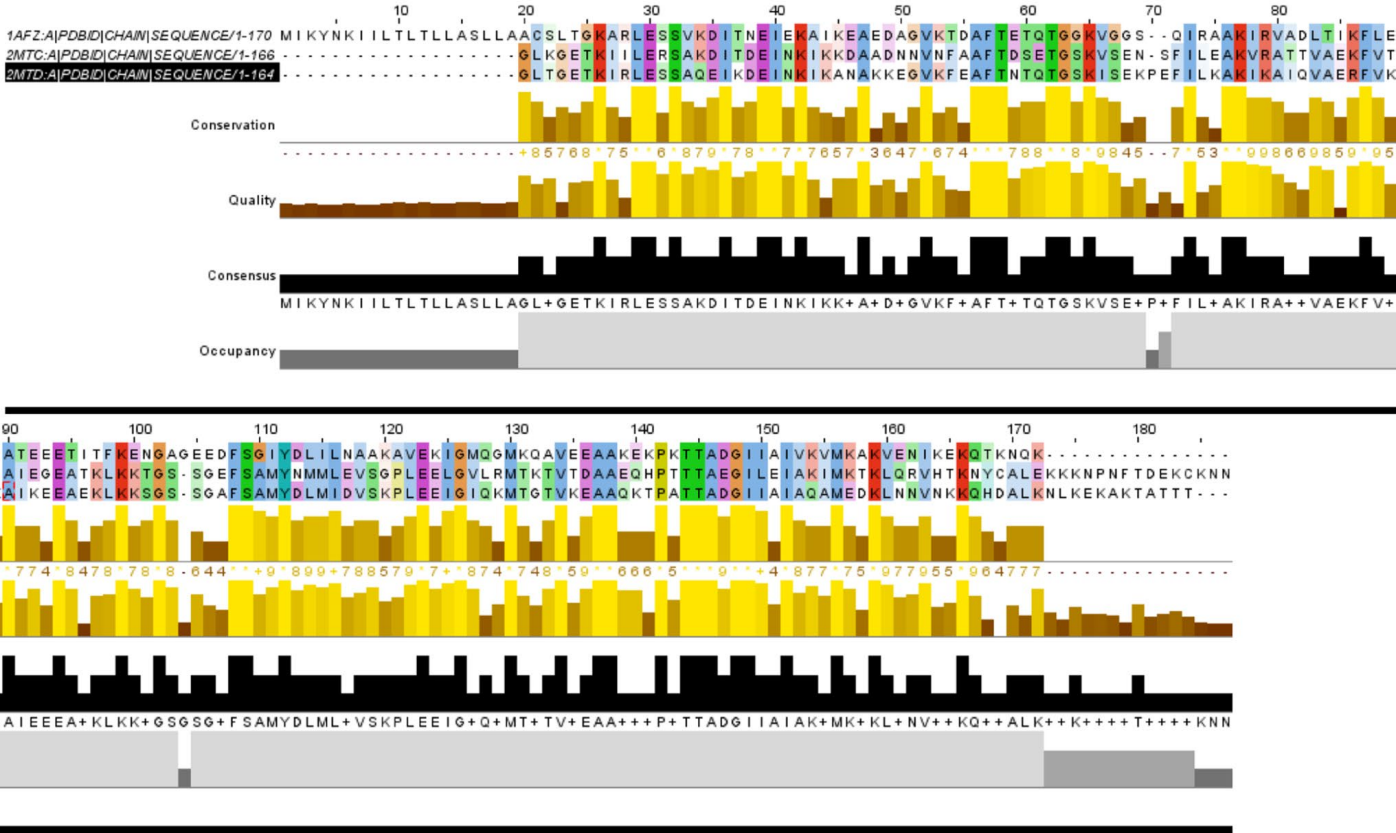
Sequence alignment of DbpA from *B. afzelii*. Comparison of sequences of *B. afzelii* DbpA (1AFZ) with homologous proteins from *B. burgdorferi* s.s. (PDB: 2MTC; Morgan and Wang, 2015) and *B. garinii* (PDB: 2MTD; Morgan and Wang, 2015)

The relaxation data derived sequence specific backbone dynamics of *B. afzelii* DbpA correlates well with the chemical shift based TALOS-N prediction and shows 5 ordered regions corresponding to alpha helical regions (Fig. 2c). No beta sheets were predicted for DbpA. The most dynamic part of the assigned backbone resonances is the longest loop (G38 – G55) which also contains a small helix. From the values of R_2_/R_1_ ratios in *B. afzelii* DbpA 32% of dynamic parts was estimated. This is in good agreement with 34% of dynamic regions found in *B. burgdorferi* s.s. DbpA (PDB: 2MTC; Morgan and Wang, 2015) and the ca. 24% of intrinsically disordered parts found in *B. garinii* DbpA (PDB: 2MTD; Morgan and Wang, 2015). These difference in dynamics are most likely linked to differences in binding mechanisms to GAGs.

In summary, we report the first characterization of DbpA from European *B. afzelii* by solution NMR spectroscopy. Backbone and side chain resonance assignments provide a crucial starting point for comparative studies of interactions between this DbpA variant and various GAG chains. Secondary structure estimates provide important first insight into structural differences among DbpA homologs that are most probably linked to their varied dissemination strategies. Backbone dynamics (and its changes) can be correlated to differential interaction mechanisms between GAG ligands and *Borrelia* DbpA variants.

## Data Availability

Set of assigned resonances is available at the Biological Magnetic Resonance Databank under accession number of 50751.
